# An explainable machine learning model for prediction of high-risk nonalcoholic steatohepatitis

**DOI:** 10.1038/s41598-024-59183-4

**Published:** 2024-04-13

**Authors:** Basile Njei, Eri Osta, Nelvis Njei, Yazan A. Al-Ajlouni, Joseph K. Lim

**Affiliations:** 1 Section of Digestive Diseases, Yale School of Medicine, New Haven, CT 06510 USA; 2 Global Clinical Scholars Research Program, Harvard Medical School, Boston, MA USA; 3 Artificial Intelligence Programme, University of Oxford Said Business School, Oxford, UK; 4 University of Texas Health San Antonio, San Antonio, TX 78229 USA; 5 Centers for Medicare and Medicaid Services, Baltimore, MD USA; 6 School of Medicine, New York Medical College, Valhalla, NY 10595 USA

**Keywords:** Hepatology, Non-alcoholic steatohepatitis

## Abstract

Early identification of high-risk metabolic dysfunction-associated steatohepatitis (MASH) can offer patients access to novel therapeutic options and potentially decrease the risk of progression to cirrhosis. This study aimed to develop an explainable machine learning model for high-risk MASH prediction and compare its performance with well-established biomarkers. Data were derived from the National Health and Nutrition Examination Surveys (NHANES) 2017-March 2020, which included a total of 5281 adults with valid elastography measurements. We used a FAST score ≥ 0.35, calculated using liver stiffness measurement and controlled attenuation parameter values and aspartate aminotransferase levels, to identify individuals with high-risk MASH. We developed an ensemble-based machine learning XGBoost model to detect high-risk MASH and explored the model’s interpretability using an explainable artificial intelligence SHAP method. The prevalence of high-risk MASH was 6.9%. Our XGBoost model achieved a high level of sensitivity (0.82), specificity (0.91), accuracy (0.90), and AUC (0.95) for identifying high-risk MASH. Our model demonstrated a superior ability to predict high-risk MASH vs. FIB-4, APRI, BARD, and MASLD fibrosis scores (AUC of 0.95 vs. 0.50, 0.50, 0.49 and 0.50, respectively). To explain the high performance of our model, we found that the top 5 predictors of high-risk MASH were ALT, GGT, platelet count, waist circumference, and age. We used an explainable ML approach to develop a clinically applicable model that outperforms commonly used clinical risk indices and could increase the identification of high-risk MASH patients in resource-limited settings.

## Introduction

Metabolic dysfunction-associated steatotic liver disease (MASLD) is the most common liver disease in the world, with a global prevalence of 25%. MASLD has an estimated prevalence of 34% in the United States. Of those with MASLD, about 20% have metabolic dysfunction-associated steatohepatitis (MASH), a more severe, progressive phenotype characterized by inflammation and damage to liver cells. Studies examining the long-term outcomes in patients with MASH suggest that the degree of fibrosis is the single most important predictor of liver-related mortality^1^. High-risk patients with MASH (i.e., those at high risk of progression to cirrhosis) are generally identified by MASLD activity score ≥ 4 and fibrosis stage ≥ 2 on liver biopsy^2^. Early identification of high-risk individuals with MASH can offer patients access to novel therapeutic options and potentially decrease the risk of cardiovascular and liver complications. However, liver biopsy, often heralded as the reference standard for diagnosis, is not always available and may be subject to sampling error.

The FibroScan-AST (FAST) score, calculated using liver stiffness measurement and controlled attenuation parameter values from transient elastography (FibroScan) and aspartate aminotransferase levels, is a validated algorithm to identify individuals with high-risk MASH^3^. However, transient elastography is not always available in resource-limited settings. Machine learning (ML) methods have shown promise in non-invasive diagnosis of MASH and advanced liver fibrosis using routine clinical data. A comparative study of various ML methods found that gradient boosting achieved the best area under the curve (AUC) scores for MASH and advanced fibrosis^4,5^. These ML models have the potential to improve MASH detection and diagnosis, ultimately leading to better management and outcomes for patients.

While ML models achieve the goal of higher predictive performance, they are uninterpretable regarding the sources of risk prediction and often fail to generalize, limiting their clinical utility. Recent advances in explainable artificial intelligence (XAI) methods can now be used to improve the interpretation, transparency, and generalizability of ML models, thus leading to more clinical decision-making confidence and more real-world adoption^6^. However, despite the promising research progress in the field, there is no clinically available XAI algorithm to identify high-risk MASH. The primary aim of our study was to apply machine learning classifiers (supervised and unsupervised) to develop a clinically applicable ML algorithm to identify high-risk MASH accurately and reliably using demographic, clinical, and laboratory data. Our secondary aim was to compare the performance of our explainable ML model with well-established clinical markers of high-risk MASH.

## Methods

### Study design and data sources

Data were derived from the National Health and Nutrition Examination Surveys (NHANES) 2017-March 2020. NHANES is a nationally representative survey of the noninstitutionalized US population conducted by the National Center for Health Statistics^7^. Data collected from 2019 to March 2020 were combined with data from the NHANES 2017–2018 cycle to form a nationally representative sample of NHANES 2017–March 2020 pre-pandemic data. We included participants aged 18 years or older who met specific inclusion criteria, such as negative Hepatitis B surface antibody, negative Hepatitis C antibody, and no history of high alcohol use (> 1 drink/day for women, > 2 drinks/day for men). To ensure the quality and consistency of data, we excluded participants with missing liver elastography data and unacceptable Vibration-Controlled Transient Elastography (VCTE) measurements^8^, such as incomplete liver elastography with less than 10 stiffness measurements and interquartile range and median ≥ 30% (Supplementary Fig. 1).

The study followed the Transparent Reporting of a Multivariable Prediction Model for Individual Prognosis or Diagnosis (TRIPOD) reporting guidelines^9^. The NHANES study protocol was approved by the NCHS research ethics review board, and participants provided written informed consent. The study was deemed exempt by the Yale institutional review board as it used publicly available deidentified data, and informed consent was waived.

### Outcome and predictors

Our outcome variable was high-risk MASLD as defined by FAST score of ≥ 0.35 and ≥ 0.67, based on previously published studies^10^. All candidate predictors were derived from the literature. The predictors included demographic information (age, sex, race, and ethnicity), physical exam findings, laboratory values, and past medical history. We additionally calculated the Homeostasis Model Assessment of Insulin Resistance [HOMA-IR = insulin (μU/mL) × fasting glucose (mmol/L)/22.5] as a surrogate marker of insulin resistance^11^. Supplementary Data lists all predictors used to train and test the models.

### Serologic biomarkers

We calculated the following serologic biomarkers of liver fibrosis at their corresponding cur-off values: Fibrosis-4 Index^12^ (FIB4; 1.30), NAFLD Fibrosis Score^13^ (NFS; -1.46), BMI-AAR-T2DM^14^ (BARD; 0.70) and Aspartate Aminotransferase to Platelet Ratio Index (APRI; 2.00)^15^. Supplementary Methods Table 1 offers details on biomarker calculations and formulas.

**Table 1:** Select clinical and laboratory characteristics of adults older than 18 years with high-risk and no high-risk MASLD with acceptable FibroScan^®^ data in the National Health and Nutrition Examination Survey between 2017 and March 2020.

Characteristic	Category	All subjects, N = 5156	No high-risk MASLD, N = 4855	High-risk MASLD, N = 301	MD	*p*
Age in years at screening		55.0 [37.0,67.0]	55.0 [37.0,67.0]	56.0 [43.0,65.0]	− 1.87	0.063043174
Gender	Female	2490 (48.3)	2393 (49.3)	97 (32.2)	–	6.52E−09
Race/Hispanic origin W/NH Asian	Not reported	528 (10.2)	479 (9.9)	49 (16.3)	–	1.14E−05
	Mexican American	511 (9.9)	473 (9.7)	38 (12.6)		
	Other Hispanic	1693 (32.8)	1590 (32.7)	103 (34.2)		
	Non-Hispanic White	1393 (27.0)	1342 (27.6)	51 (16.9)		
	Non-Hispanic Black	788 (15.3)	749 (15.4)	39 (13.0)		
	Non-Hispanic Asian	243 (4.7)	222 (4.6)	21 (7.0)		
Diabetes mellitus type 2	Yes	609 (11.8)	527 (10.9)	82 (27.2)	–	4.16E−14
Body mass index (kg/m**2)	–	28.2 [24.5,33.0]	28.0 [24.3,32.5]	34.2 [29.4,39.0]	− 13.09	1.50E−28
Waist circumference (cm)	–	98.7 [88.0,110.0]	97.9 [87.4,108.8]	113.0 [101.7,123.8]	− 14.13	6.66E−32
Aspartate aminotransferase (AST) (U/L)	–	19.0 [16.0,23.0]	19.0 [16.0,22.2]	36.0 [28.0,49.0]	− 17.83	8.03E−42
Alanine aminotransferase (ALT) (U/L)	–	17.8 [13.0,25.0]	17.0 [13.0,23.0]	46.0 [31.0,62.0]	− 19.27	7.75E−46
Gamma glutamyl transferase (GGT) (IU/L)	–	21.0 [14.0,30.0]	20.0 [14.0,28.4]	43.0 [28.0,75.0]	− 12.15	2.95E−25
Albumin, refrigerated serum (G/dL)	–	4.1 [3.9,4.3]	4.1 [3.9,4.3]	4.1 [3.8,4.3]	− 0.01	–
Platelet count (1000 cells/UL)	–	237.0 [201.0,279.0]	238.0 [202.0,279.0]	219.0 [186.0,262.0]	− 15.85	–
Glycohemoglobin (%)	–	5.6 [5.3,6.0]	5.6 [5.3,6.0]	6.0 [5.5,6.9]	− 7.41	4.35E−12
Fasting glucose (mg/dL)	–	105.6 [98.0,117.5]	105.0 [97.6,116.2]	117.4 [105.0,142.4]	− 7.67	9.28E−13
Insulin (μU/mL)	–	10.7 [7.1,15.9]	10.4 [7.0,15.2]	20.4 [13.0,29.1]	− 13.29	9.07E−29
Direct HDL-cholesterol (mg/dL)	–	50.0 [42.0,60.0]	51.0 [43.0,60.0]	43.0 [36.0,50.0]	11.84	3.80E−25
Homair	–	2.9 [1.9,4.6]	2.8 [1.8,4.4]	6.2 [3.8,9.8]	− 12.42	4.07E−26

High-Risk MASLD defined as subjects with a FAST score cutoff at ≥ 0.35. Further, *p*-values calculated using appropriate statistical tests based on distribution and variance. Continuous variables shown as median [IQR]; categorical variables as count (%). MD, mean difference.

### Development of a machine learning model using XGBoost

We used the eXtreme Gradient Boosting (XGBoost) algorithm to develop our machine learning model. The data was split into three independent cohorts of patients with approximately equal proportions of subjects with high-risk MASLD: one for training the model (training set), another for validation during hyperparameter optimization (validation set), and a test or holdout set (test set) to evaluate the prediction performance. We performed 100 iterations of model training with hyperparameter optimization to maximize the harmonic mean of sensitivity, specificity, positive predictive value (PPV), and negative predictive value (NPV), a custom performance metric developed to maximize all individual metrics and penalize for outliers with low metrics. After training, we tested the model on the independent test set. The evaluation metrics we used included the area under the receiver operating characteristic curve (AUROC), accuracy, sensitivity, specificity, PPV, and NPV. To estimate the 95% confidence intervals (CI) for these metrics, we used a bootstrapping method with 1000 iterations. We also performed k-fold cross-validation to evaluate model robustness and to reduce bias from data partitioning. Details about model training, hyperparameter optimization, and strategies to minimize overfitting on Supplementary Methods 2.

### Shapley additive explanations

To facilitate the interpretation of our XGBoost classification model, we used Shapley Additive Explanations (SHAP) approach^16,17^ to determine the contribution of each predictor, known as a feature in computer science, toward the final prediction of high-risk MASLD. SHAP values provide a measure of predictor importance that accounts for both the individual feature values and their interactions with other features based on their impact on the ultimate prediction.

### Missing data handling

We did not censor patients with missing data or impute missing data to train and evaluate the XGBoost models due to their unique ability to learn and inference predictions despite data missingness. However, other model algorithms (e.g., logistic regression and random forest) are unable to handle missing data and thus we used a K nearest-neighbor (KNN) imputed^18^ dataset for both training and evaluation. We compared prediction performance between XGBoost, logistic regression, and random forest on a KNN-imputed dataset to compare diagnostic performance on models trained on the same dataset (Supplementary Table 1).

### Statistical analysis

We used χ^2^ and t-tests to describe patient characteristics between the high-risk MASLD outcome groups in the complete set as well as training and test sets. We included demographics, clinical and laboratory information, the number of participants, and missing data. Additionally, we report mean differences between high-risk NASH groups. For continuous variables, we used the Shapiro normality test and Levene equal variance test to determine the appropriate statistical test (independent t-test, bootstrapped comparison of means, Welch’s t-test, or Yuen’s trimmed t-test) to compare means and generate CIs. For categorical variables, we tested binary variables with Fisher’s exact test and multiclass variables with chi-square (χ^2^).

### NCHS ethics statement

The research presented in this paper adheres to the ethical principles and guidelines set forth by the National Center for Health Statistics (NCHS). The NCHS is committed to ensuring the rights, welfare, and privacy of individuals participating in research studies. The NCHS ethics statement underscores our commitment to upholding the highest ethical standards in research. By adhering to these principles, we aim to contribute to the advancement of knowledge while ensuring the protection and well-being of all participants involved in our study.

For further information about the NCHS ethics guidelines, please refer to the official documentation available at: https://www.cdc.gov/nchs/data/ahcd/nhamcs_erb_letter_2016.pdf.

## Results

### Subject characteristics

There was a total of 5156 subjects meeting the inclusion criteria. The prevalence of high-risk MASLD at FAST ≥ 0.35 and FAST ≥ 0.67 were 5.8% and 1.1%, respectively. The median age was 55 (IQR range 37–67 years), and 2490 (48%) were women among all subjects (Table 1). There were more men than women in the high-risk MASLD group (67.5%, p < 0.001) and more Hispanic individuals (12.6%, p < 0.001) in the high-risk MASLD compared to the no high-risk MASLD group (9.7%). The high-risk MASLD group had a higher prevalence of diabetes in their medical history (27.2%, p < 0.001) compared to the no high-risk MASLD group. Physical exam results showed higher body mass index (BMI, median 34 kg/m^2^, p < 0.001) and waist circumference (median 113 cm, p < 0.001) measurements for the high-risk MASLD subjects. In terms of laboratory results, the high-risk MASLD group had higher liver enzymes (median AST, ALT, GGT of 36, 46, 43 U/L, respectively, p < 0.001), lower platelet counts (median 219 × 103 cells/μL), and higher hemoglobin (Hb)A1c (median 6.0, p < 0.001), plasma glucose (median 117 mg/dL, p < 0.001), and insulin (20.4 μU/mL, p < 0.001). Additionally, the high-risk MASLD subjects had lower levels of HDL (median 43 mg/dL, p < 0.001). A complete comparison of all 127 predictors used to develop the exploratory ML models (i.e., prior to selecting the top 5 predictors to fine-tune subsequent models) can be found in Supplementary Data.

### Predictive performance OF XGBoost MASLD models

**Figure 1:**
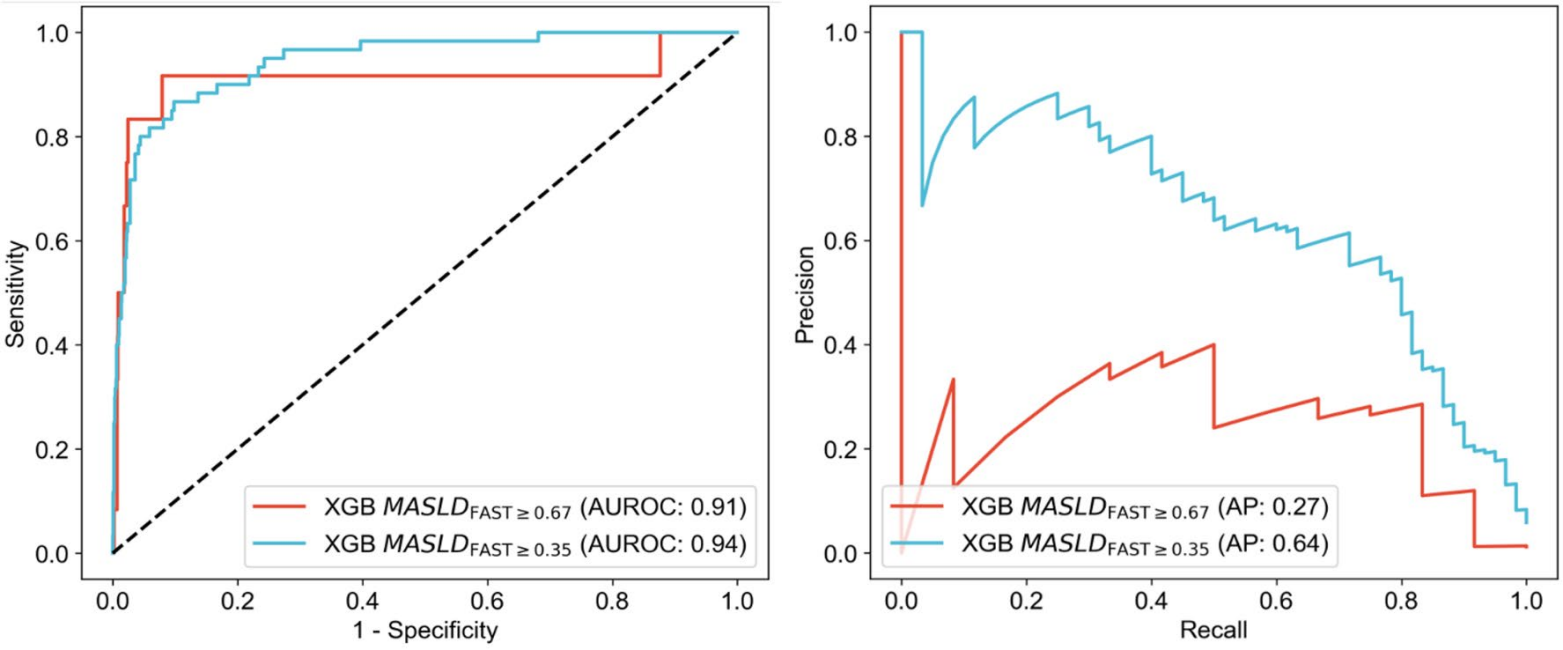
AUROC and PR curves for XGBoost MASLD models tested on holdout dataset. AUROC and PR curves for two trained, optimized, and internally validated gradient-boosting ML models to predict high-risk MASLD at FAST ≥ 0.35 and 0.67, XGBoost MASLDFAST ≥ 0.35 (blue) and XGBoost MASLDFAST ≥ 0.67 (red), respectively. Both models were trained on five clinical predictors (Nvar = 5) including ALT, GGT, platelets, age, and BMI. *XGBoost (XGB)* eXtreme Gradient Boosting, *AUROC* area under the receiving operator characteristic, *PR* precision-recall, *AP* average precision.

We trained and optimized multiple hyperparameters of XGBoost MASLD models fine-tuned on the top 5 predictors, and at FAST ≥ 0.35 and 0.67. Next, we evaluated the prediction performance on the test set (i.e., holdout set) and found a high AUROC across all XGBoost MASLD models, ranging from 0.91 to 0.97 (Fig. 1; Supplementary Fig. 2). Further, for XGBoost models trained on the top 5 predictors, k-fold cross-validation at fivefold showed ranges of cross-validated AUROC of 0.92 to 0.92 and 0.51 to 0.99 for XGBoost MASLD_FAST≥0.35_ and XGBoost MASLD_FAST≥0.67_, respectively. Further, cross-validated PR curves ranged from 0.56 to 0.82 and 0.05 to 0.47 (Supplementary Fig. 3). In addition, bootstrapped evaluations of performance metrics of XGBoost MASLD_FAST≥0.35_ showed a mean (95% CI) of AUROC with 0.95 (0.91–0.97), accuracy with 0.95 (0.94–0.97), sensitivity with 0.71 (0.59–0.83), specificity with 0.97 (0.96–0.98), PPV with 0.59 (0.47–0.70), and NPV with 0.98 (0.97–0.99). In addition, XGBoost MASLD_FAST≥0.67_ showed AUROC with 0.91 (0.77–0.99), accuracy with 0.98 (0.97–0.99), sensitivity with 0.50 (0.20–0.80), specificity with 0.99 (0.98–0.99), PPV with 0.30 (0.11–0.50), and NPV with 0.99 (0.99–1.00) (Supplementary Fig. 4). We compared other classical ML models, including logistic regression (LR) and random forest (RF), to XGBoost MASLD models. Unlike XGBoost, LR and RF do not support missing data; therefore, we imputed missing data using KNN imputation in the training, validation, and test sets. We trained models on data with all 127 predictors or 5 predictors, and at FAST ≥ 0.35 or 0.67. We used the harmonic mean of the accuracy, AUROC, sensitivity, specificity, PPV, and NPV to rank models by their performance. XGBoost was the top-performing model in 3 of the 4 comparisons, followed by LR and RF both with 2 out of 4 (Supplementary Table 1).

### Comparing serologic biomarkers and XGBoost MASLD models

We compared well-established serologic biomarkers of liver fibrosis to XGBoost MASLD_FAST≥0.35_, and found our model performed with the highest AUROC at 0.94 while FIB4 (≥ 1.30), NFS (≥ − 1.46), BARD (≥ 2.00), and APRI (≥ 0.70) performed at AUROC 0.50, 0.54, 0.39, 0.50, respectively. While FIB, NFS, and APRI had the highest sensitivity and NPV at 1.0, XGBoost MASLD_FAST≥0.35_ had a sensitivity of 0.77 and NPV of 0.99. In addition, XGBoost MASLD_FAST≥0.35_ had the highest specificity and PPV at 0.97 and 0.61 while other serologic biomarkers did not surpass 0.14 and 0.06, respectively (Table 2).

**Table 2:** Performance of serologic biomarkers and XGB MASLD_FAST≥0.35_ model.

Biomarker (cut-off)	Accuracy	Sensitivity	Specificity	PPV	NPV	AUROC
FIB4 (1.30)	0.06	1.00	0.00	0.06	1.00	0.50
NFS (− 1.46)	0.14	1.00	0.08	0.06	1.00	0.54
BARD (2.00)	0.17	0.65	0.14	0.04	0.87	0.39
APRI (0.70)	0.06	1.00	0.00	0.06	1.00	0.50
XGB MASLD_FAST≥0.35_ (*p* ≥ 0.50)	0.95	0.77	0.97	0.61	0.99	0.94

Comparison of prediction performance of serologic biomarkers and AI models in identifying MASLD defined as FAST score ≥ 0.35. Biomarkers include FIB4, NFS, BARD, APRI, with XGB MASLD_FAST≥0.35_. Serologic biomarkers and cut-off values: FIB4, Fibrosis-4 Index; NFS, NAFLD Fibrosis Score; BARD, BMI-AAR-T2DM; APRI, Aspartate Aminotransferase to Platelet Ratio Index. *p*, probability of predicting high-risk MASLD; AAR, AST/ALT ratio. $FIB4=(Age\times AST)/(Plt\times \sqrt{ALT})$, $NFS=Age\times 0.037+BMI\times 0.094+DM\times 1.13+AAR\times 0.99-Plt\times 0.013-Albumin\times 0.66$, $BARD=1\times \left(BMI\ge 28\right)+2\times \left(AAR\ge 0.8\right)+1\times DM$, $APRI=(AST\times 40)/Plt$, XGBoost model trained on ALT, GGT, platelets, age, and BMI to predict FAST ≥ 0.35.

### Explaining cohort- and patient-level predictions by XGBoost MASLD models

**Figure 2:**
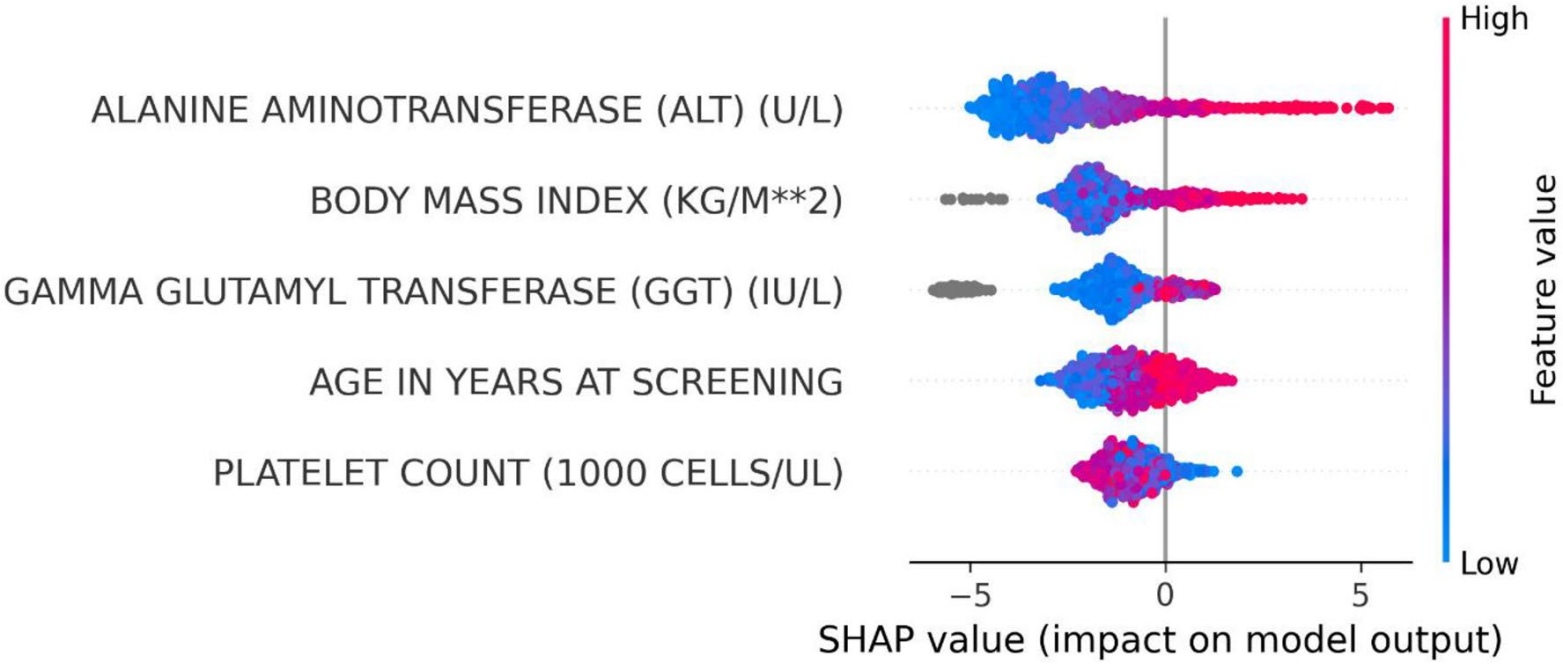
Impact of predictors on high-risk MASLD prediction using SHAP values. Summary Shapley Additive Explanations (SHAP) plot shows the importance and impact of various training variables (predictors) on XGB MASLD_FAST≥0.35_. The SHAP values on the x-axis quantify the influence of each predictor, with positive values favor high-risk MASLD prediction and negative values favor no high-risk MASLD prediction. The predictors, including ALT, BMI, GGT, age, and platelet count, are ranked by magnitude of impact. Predictor values are color-coded, with red indicating higher values and blue lower variable values (e.g., ALT of 120 U/L in red and 12 U/L in blue).

We conducted SHAP for tree-based models to evaluate how the predictors used to train the models influenced the predictions made by XGBoost MASLD models (Fig. 2; Supplementary Figs. 5–6). A prediction of high-risk MASLD was more likely when SHAP > 0 (i.e., prediction probability, *P*_pred_ ≥ 0.50), and a prediction of *no* high-risk MASLD was more likely when SHAP < 0 (*P*_pred_ < 0.50).

### Explaining cohort-level XGBoost MASLD predictions

In descending order of impact on predictions by XGBoost MASLD_FAST≥0.35_ trained on the top 5 predictors, ALT, BMI, GGT, age, and platelet counts. ALT, BMI, GGT, age had a positive impact on high-risk MASLD prediction at FAST ≥ 0.35, whereas platelet count had a negative impact on high-risk MASLD prediction (i.e., positive impact on *no* high-risk MASLD prediction at FAST ≥ 0.35). Further, predictions by XGBoost MASLD_FAST≥0.67_ were influenced first by ALT, followed by BMI, GGT, platelet count, and age at last. ALT, BMI, GGT had a positive impact on high-risk MASLD prediction at FAST ≥ 0.67, platelet count had a negative impact, and any age had a negative impact on predictions.

We show the top 20 predictor contributions for XGBoost MASLD models trained on all 127 predictors (Supplementary Figs. 5–6). ALT, GGT, BMI were the top 3 predictors for both XGBoost MASLD_FAST≥0.35_ and XGBoost MASLD_FAST≥0.67._ Platelet count was in the top 20 features of both models, whereas age was only in XGBoost MASLD_FAST≥0.35_.

### Distribution of predictor values, model contribution, and prediction accuracy

**Figure 3:**
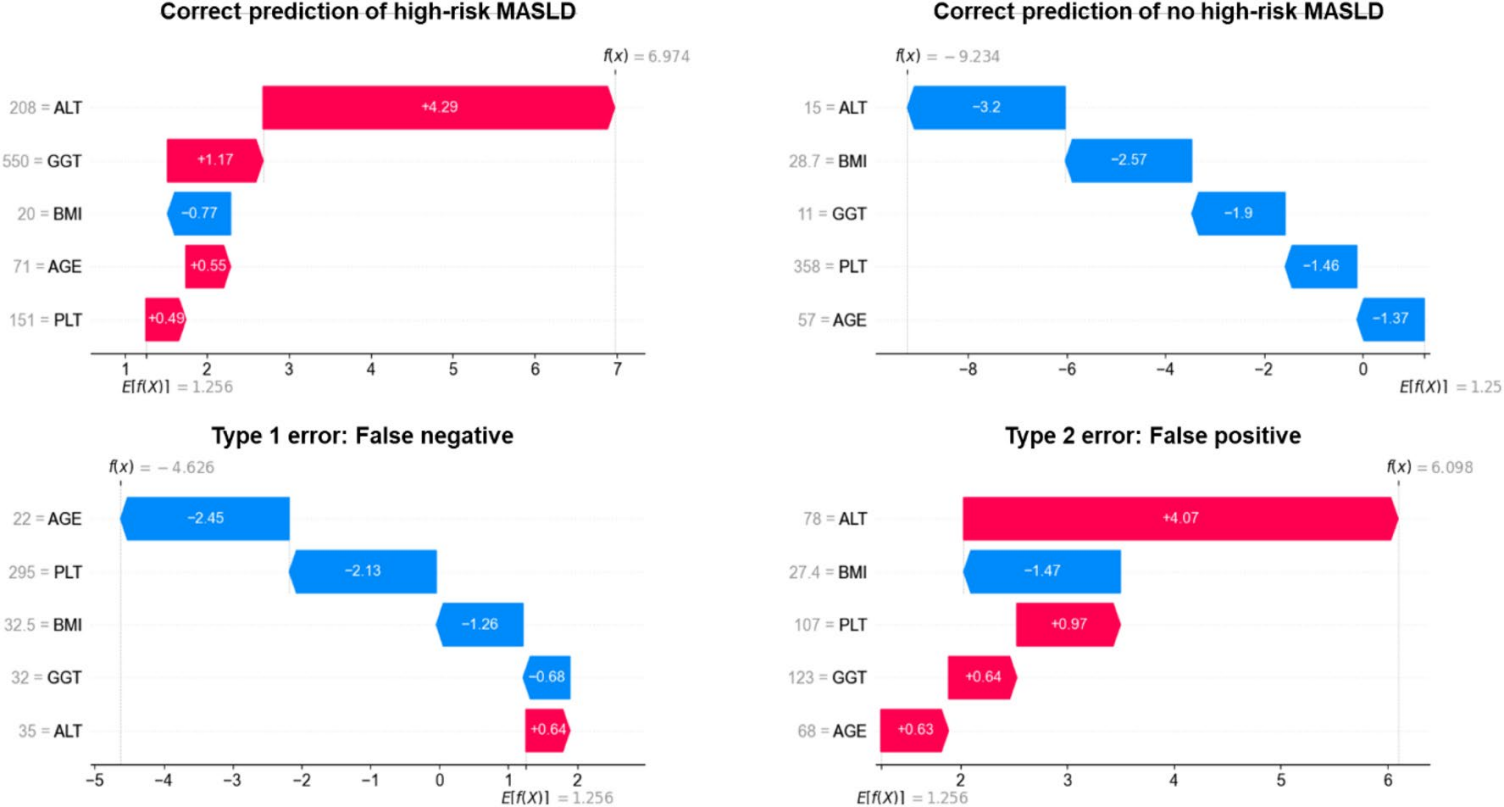
Patient-Specific SHAP Value Impact on XGB MASLD_FAST≥0.35_ Predictions. Four cases of patients and their corresponding local SHAP values showing the contribution of ALT, GGT, platelets, age, and BMI on the predictions by XGB MASLD_FAST≥0.35_. Upper panel shows correct high-risk MASLD (top left) and no high-risk MASLD (top right) predictions. Bottom panel shows incorrect predictions, including a false negative (bottom left) and false positive (bottom right). Red and blue bars indicate attributes driving the model to predict high-risk MASLD and no high-risk MASLD, respectively. Each subplot provides specific patient data points, their contribution to the model output (f(x)), and the baseline expected value (E[f(X)]) of the model.

We compared the predictor-specific SHAP values (i.e., contribution to model prediction by that unique predictor) and their corresponding predictor values for each subject in the test set (Supplementary Fig. 7). Generally, there was a positive correlation between ALT, GGT, and BMI and their SHAP values, a negative correlation between platelet count and its SHAP values, and a positive correlation between age and its corresponding SHAP values for XGBoost MASLD_FAST≥0.35_ only. We display the few false positive (orange) and false negative predictions (green), and further investigate unique cases of inaccurate predictions in Fig. 3.

### Explaining patient-level XGBoost MASLD predictions

We describe four cases of unique subjects and their corresponding local SHAP values showing the contribution of ALT, GGT, platelets, age, and BMI on the predictions by XGB MASLD_FAST≥0.35_ (Fig. 3).

The correct prediction of high-risk MASLD was primarily driven by an ALT of 208 U/L (SHAP_ALT_ =  + 4.29), followed by a GGT of 550 U/L (SHAP_GGT_ =  + 1.17), age of 71 years old (SHAP_AGE_ =  + 0.55), and a platelet count of 151 × 1000 cells/µL (SHAP_PLATELETS_ =  + 0.49), whereas a BMI of 20 kg/m^2^ had a negative impact (SHAP_BMI_ = − 0.77) on high-risk MASLD prediction. In total, the sum of individual SHAP contributions plus the model’s baseline expected value ($E\left[f\left(X\right)\right]=1.256$) yields a total SHAP ($f\left( X \right)$) = $1.256 + 4.29 + 1.17 + 0.55 + 0.49 {-} 0.77 = + 6.975$. Further, the correct prediction of *no* high-risk MASLD was primary driven by an ALT = 15 U/L (− 3.2), BMI = 28.7 kg/m^2^ (− 2.57), GGT = 11 U/L (− 1.9), platelet count of 358 × 1000 cells/µL (− 1.46), and age of 57 years old (− 1.37), yielding a total SHAP equal to − 9.234.

On the other hand, a false negative prediction of high-risk MASLD for a specific subject was driven by their age of 22 (− 2.45), platelet count of 295 × 1000 cells/µL (− 2.13), BMI of 32.5 kg/m^2^ (− 1.26), GGT of 32 U/L, ALT of 35 U/L (+ 0.64), all totaling to $f\left(X\right)=-4.626$, thus favoring a prediction of *no* high-risk MASLD. Further, a false positive prediction for another subject was driven by their ALT of 78 U/L (+ 4.07), BMI of 27.4 kg/m^2^ (− 1.47), platelet count of 107 × 1000 cells/µL (+ 0.97), GGT of 123 U/L (+ 0.64), and age of 68 (+ 0.63), all totaling to $f\left(X\right)=+6.098$, thus favoring a prediction of high-risk MASLD.

## Discussion

In this observational study, we developed an ensemble-based machine learning using XGBoost to detect individuals in the U.S. population with high-risk MASLD and explored cohort- and subject-level prediction interpretability using explainable artificial intelligence with SHAP analysis. This application of explainable machine learning for the identification of high-risk MASLD contributes to the growing body of research in this area. To the best of our knowledge, this is the first nationwide application of explainable machine learning for the identification of high-risk MASLD. While traditional machine learning applications have shown promise in detecting MASLD, MASH and advanced fibrosis, our explainable XGBoost MASLD model demonstrates unique functionalities not previously reported in the literature, including: (1) ability to learn despite missing data, (2) use of five, easily accessibly patient features (ALT, GGT, platelet count, age, and BMI), and (3) prediction explanation with feature-specific SHAP values.

Although prior applications of traditional machine learning to detect MASLD, MASH and advance fibrosis have produced promising results, we showed that our explainable XGBoost MASLD model outperformed previous traditional ML models in detecting high-risk MASLD. Compared to Wu et al.^19^, Docherty et al.^4^, and Ghandian et al.^5^, who reported sensitivities up to 0.82 and specificities up to 0.79, our model achieved comparable sensitivity (mean [95%], 0.71 [0.59–0.83]), but higher specificity (0.97 [0.96–0.98]), accuracy (0.95 [0.94–0.97]), and AUROC (0.95 [0.91–0.97]). Additionally, we report a good PPV of 0.59 [0.47–0.70] and excellent NPV of 0.98 [0.97–0.99]. This is a significant improvement in performance, which can help to improve the diagnosis and management of high-risk MASLD patients in resource-limited settings. It is important to acknowledge, however, that the natural history of MASLD is complex and the terminology describing disease stages is evolving. Additionally, our study's endpoints might differ from those in previous studies, which could impact comparative assessments. Therefore, our findings contribute to a broader understanding of the potential of machine learning in this evolving field and must be interpreted within this context.

Furthermore, we also compared the performance of serologic biomarkers and the XGBoost MASLD in identifying high-risk MASLD patients. Although FIB4, NFS, and APRI had higher sensitivity and NPV, XGBoost MASLD had a good sensitivity of 0.77 and excellent NPV of 0.99 in the test set. In addition, XGBoost MASLD had the highest specificity and PPV at 0.97 and 0.61 while other serologic biomarkers did not surpass 0.14 and 0.06, respectively. These results suggest that our XGBoost model can serve as a promising tool for identifying high-risk MASLD patients.

Our findings reveal that the subject characteristics in our study are consistent with the classical clinical manifestation of the MASLD-MASH disease spectrum. We observed that the high-risk MASLD group had higher rates of obesity, type 2 diabetes, and metabolic syndrome, which includes hypertriglyceridemia, low HDL-C, and abdominal obesity^20^, when compared to the non-high-risk group. Additionally, the high-risk MASLD group demonstrated elevated transaminases in the absence of heavy alcohol consumption and no history of hepatitis B or C, which aligns with the diagnostic criteria for MASLD. These results are consistent with previous studies that have identified obesity and type 2 diabetes as significant risk factors for the development of MASLD^21^.

This study had several limitations. First, although the optimized XGBoost model was validated using a test (holdout) set, it would need to be further prospectively externally validated before widespread adoption. Second, we did not have data on liver biopsy for gold-standard comparison. Third, the adoption of the optimized model in clinical practice as well as its integration into electronic medical records will need to be evaluated in future studies. Fourth, as with any machine learning modality, possible “overfitting” is a significant limitation. To address this, we performed the following overfitting mitigating strategies: (1) using 3 sets of data partitioning to validate the model while training and a separate test (holdout) set for internal validation, (2) hyperparameter optimization through 100 iterations of XGBoost MASLD models with unique combinations of regularization, subsampling parameters, (3) early stopping in model training, (4) balanced weighting to avoid overfitting on a highly prevalent class, (5) and internal validation strategies including k-fold cross-validation and bootstrapping metrics on the test set. Finally, a major limitation that warrants discussion is the dependency of the XGBoost model performance on the training cohort characteristics. This raises important concerns about the generalizability of these models in other settings and populations. The difficulty in direct comparison of ML models unless developed and validated on similar cohorts underscores the necessity for cautious application and interpretation of our findings across different clinical environments. This limitation highlights the importance of ongoing evaluation and adaptation of ML models in diverse settings to ensure their relevance and efficacy. Finally, given concerns with generalisability, future research is advised to utilize an external cohort for validation, which was out of the scope for this work but would significantly support the generalisability of the conclusion(s) made.

## Conclusion

In conclusion, our study demonstrates the potential of explainable machine learning in the detection of high-risk MASH. The development of an XGBoost model that outperforms well-established serologic tests has shown the ability of machine learning to detect high-MASH in a more comprehensive and flexible manner. The high complexity of our model allows for the detection of heterogeneous subphenotypes, a feature not present in most serologic tests. While the pathophysiology of liver fibrosis in MASH is complex and variable, our model has proven successful in classification. These findings suggest that a more multidisciplinary approach that incorporates machine learning may lead to improved diagnosis and management of patients with MASH, ultimately optimizing clinical outcomes. Further studies are needed to explore the clinical applications of our proposed XGBoost model in identifying high-risk MASH patients. If externally validated, our explainable ML model could be used to increase the identification of high-risk MASH patients in resource-limited settings.

### Supplementary Information

Supplementary Information 1.

Supplementary Information 2.

Supplementary Information 3.

Supplementary Information 4.

## Data Availability

All data associated with processing is published with this manuscript. NHANES data is publicly available online. The data that support the findings of this study are publicly available data, from the National Health and Nutrition Examination Surveys (NHANES). However, restrictions apply to the availability of these data, which were used under license for the current study, and so are not publicly available. Data is however available from the authors upon reasonable request and with permission of NHANES. The corresponding author can be contacted for data requests.

## Code availability

Available upon reasonable request made to the authors.
