# Supplementary material for: An explainable machine learning model for prediction of high-risk nonalcoholic steatohepatitis
Source: Sci Rep. 2024 Apr 13;14:8589. doi: 10.1038/s41598-024-59183-4 (PMC11016071; doi:10.1038/s41598-024-59183-4)
Supplement: Supplementary file 3 — Supplementary Information 3. [file 41598_2024_59183_MOESM3_ESM.docx]

**Supplemental Figures**


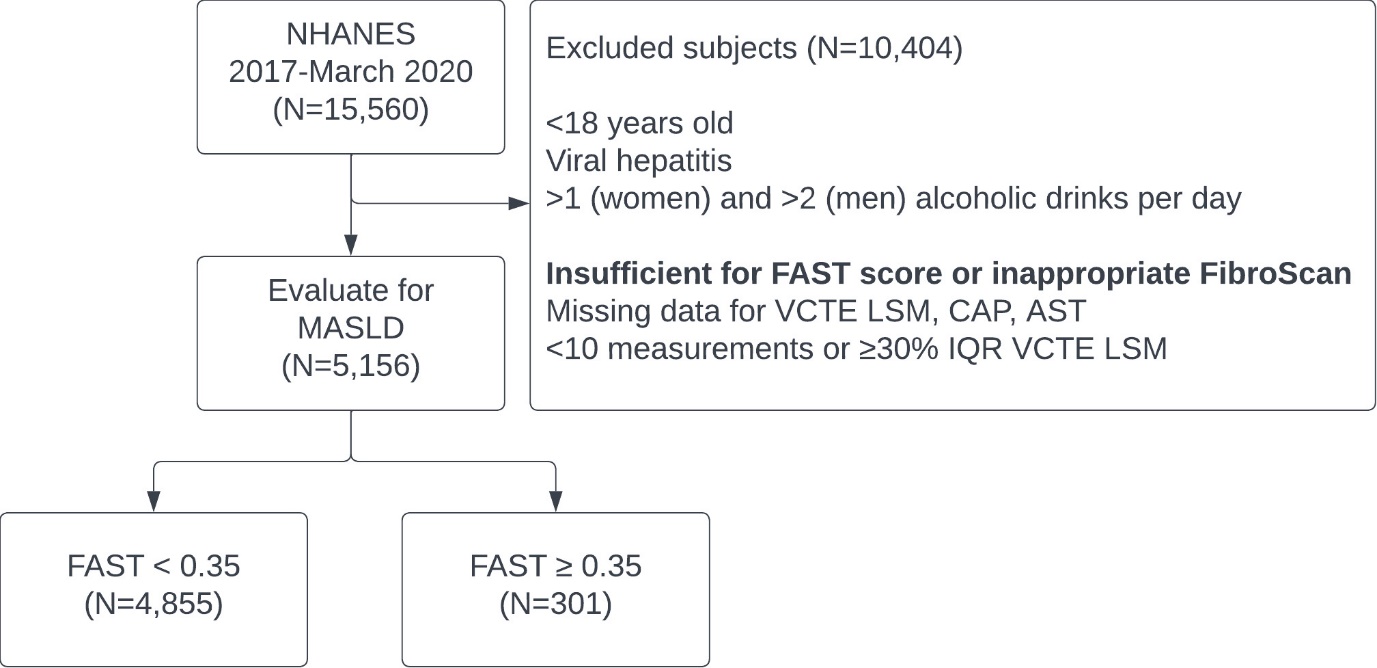


**Supplemental Figure 1. Subject Selection Flowchart from NHANES 2017-March 2020 Cohort.** Subjects under 18, with viral hepatitis, or excessive alcohol intake (>1 drink/day for women, >2 for men) were excluded. Further, those with insufficient data for FAST score calculation or inappropriate FibroScan studies (missing VCTE LSM, CAP, AST, <10 measurements, or ≥30% IQR in VCTE LSM) were removed. The final analysis included 5,156 subjects, stratified by FAST score into low-risk (<0.35) and high-risk (≥0.35) for MASLD.


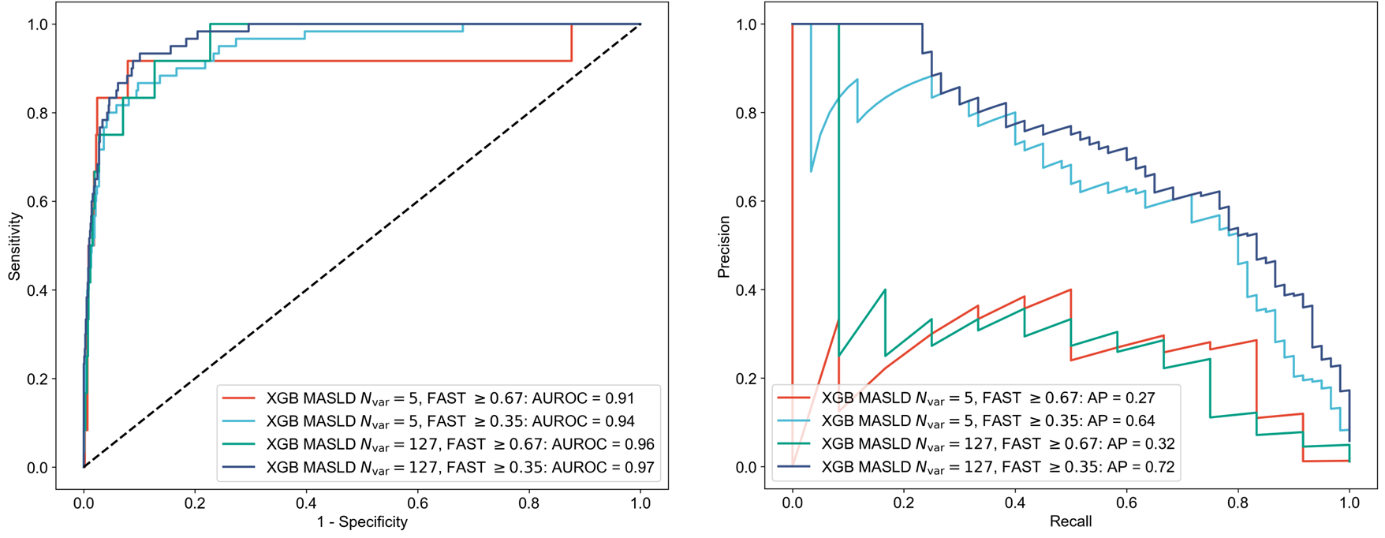


**Supplemental Figure 2. Performance Metrics on Holdout Test Set of Multiple XGB MASLD models.**


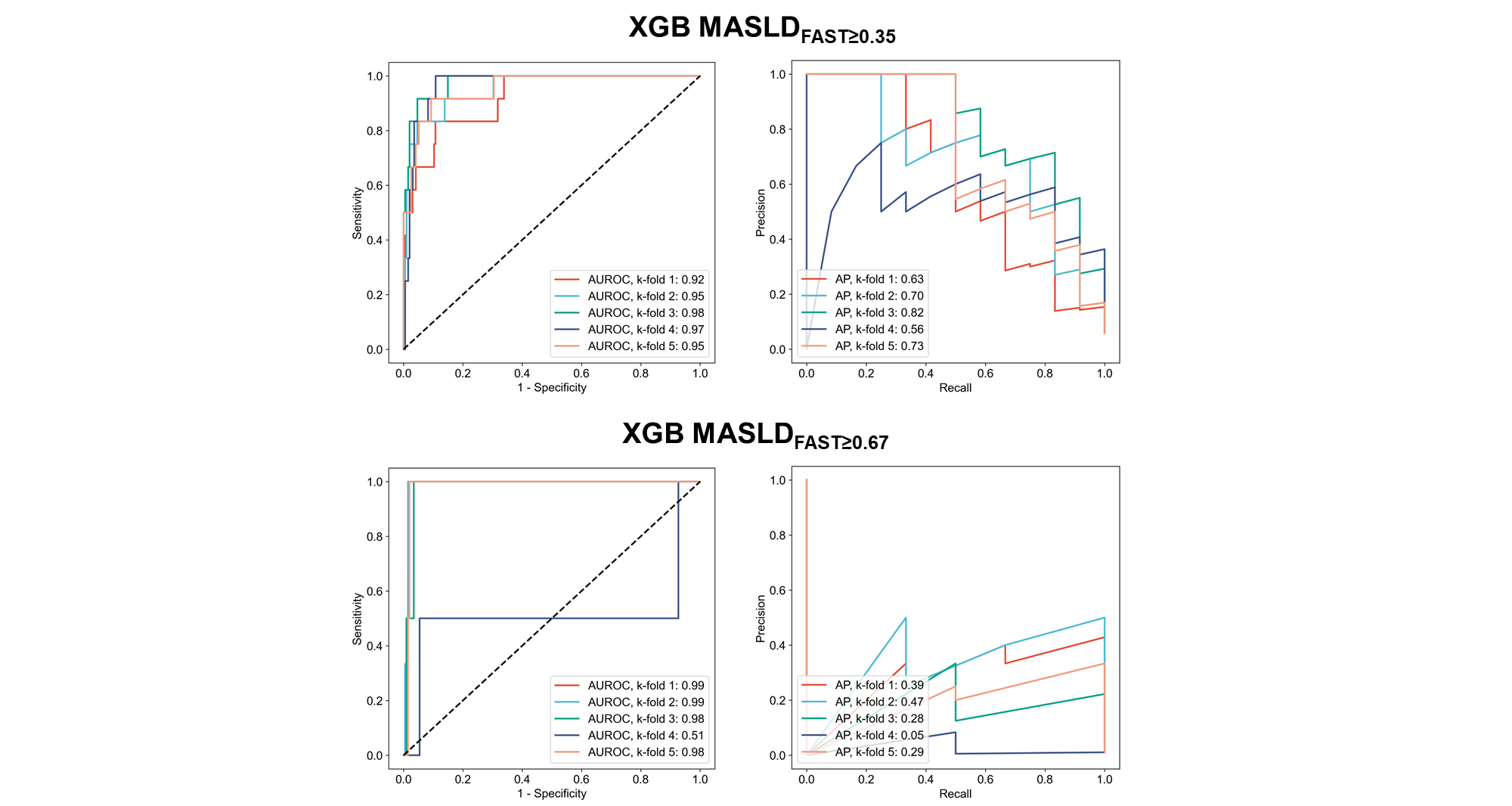


**Supplemental Figure 3. 5-Fold Cross-Validation Performance Metrics.** 5-fold cross-validation on the holdout test set for XGB MASLD models trained on top 5 predictors. AUROC curves (left) and PR curves (right) for each fold. The top panel shows results for the first set of folds, while the bottom panel presents a separate set. PR, precision-recall; AP, average precision; top 5 predictors: ALT, GGT, platelets, age, BMI.


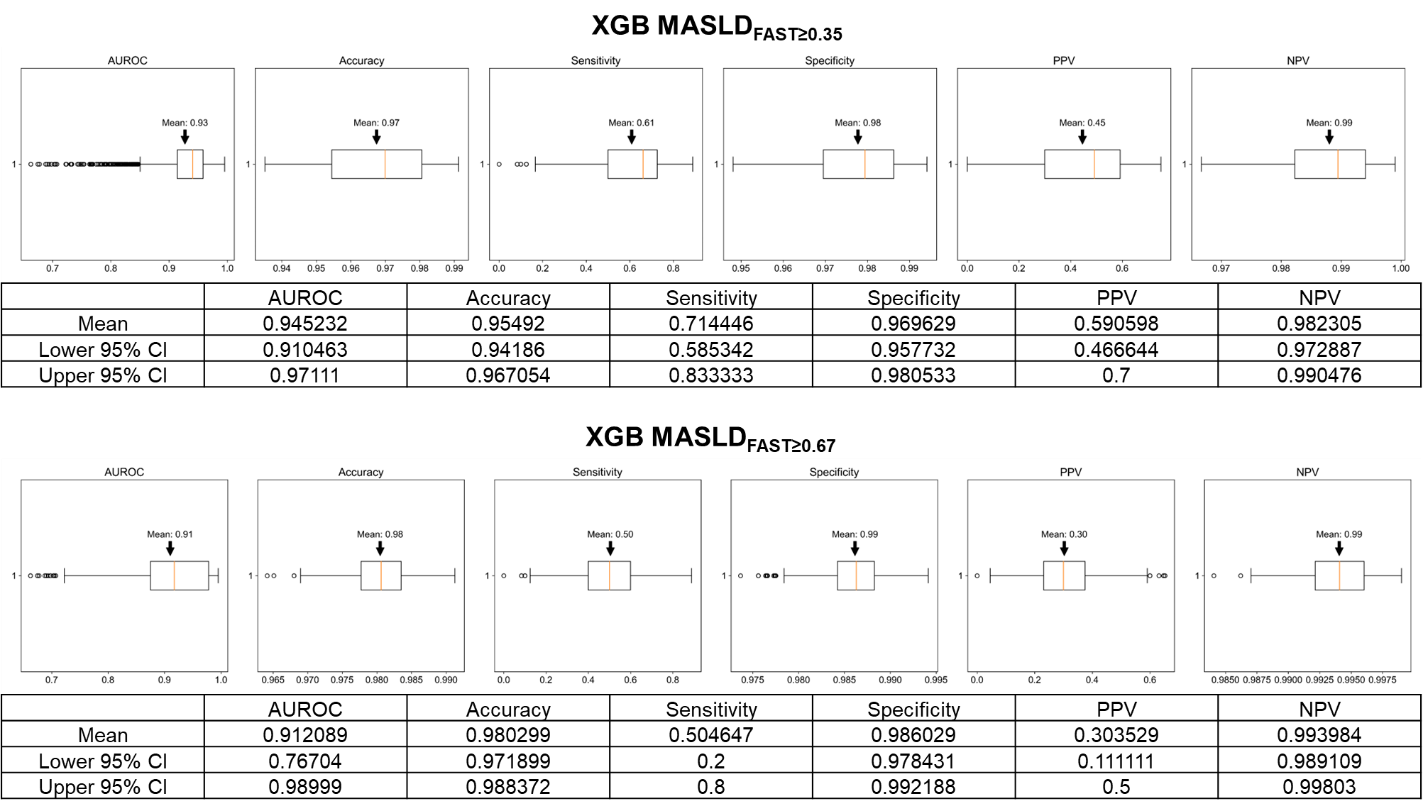


**Supplemental Figure 4.** Box plots comparing the performance metrics of the XGB MASLD model at thresholds ≥0.35 (top) and ≥0.67 (bottom). Metrics shown are AUROC, accuracy, sensitivity, specificity, PPV, and NPV, derived from 1000 bootstrapped iterations on the test dataset. The plots show the median, IQR, and mean values, with the mean marked by arrows. Tables correspond to bootstrapped mean metrics and 95% CI.

| N_var_ | FAST  cut-off | Model | Accuracy | AUROC | Sensitivity | Specificity | PPV | NPV | H_mean_ |
| --- | --- | --- | --- | --- | --- | --- | --- | --- | --- |
| 5 | 0.35 | Logistic Regression | 0.96 | 0.97 | 0.4 | 1 | 0.86 | 0.96 | 0.771501 |
| 5 | 0.35 | Random Forest | 0.97 | 0.95 | 0.52 | 1 | 0.91 | 0.97 | 0.840752 |
| 5 | 0.35 | XGBoost | 0.954 | 0.943 | 0.633 | 0.974 | 0.603 | 0.977 | 0.811134 |
| 5 | 0.67 | Logistic Regression | 0.99 | 0.99 | 0.17 | 1 | 1 | 0.99 | 0.54982 |
| 5 | 0.67 | Random Forest | 0.99 | 0.95 | 0.25 | 1 | 0.75 | 0.99 | 0.637879 |
| 5 | 0.67 | XGBoost | 0.98 | 0.958 | 0.583 | 0.984 | 0.304 | 0.995 | 0.660046 |
| 127 | 0.35 | Logistic Regression | 0.96 | 0.97 | 0.45 | 0.99 | 0.79 | 0.97 | 0.7893 |
| 127 | 0.35 | Random Forest | 0.96 | 0.97 | 0.25 | 1 | 0.94 | 0.96 | 0.653731 |
| 127 | 0.35 | XGBoost | 0.958 | 0.969 | 0.733 | 0.972 | 0.62 | 0.983 | 0.845178 |
| 127 | 0.67 | Logistic Regression | 0.99 | 0.95 | 0.25 | 1 | 0.5 | 0.99 | 0.595662 |
| 127 | 0.67 | Random Forest | 0.99 | 0.97 | 0 | 1 | N/A | 0.99 | N/A |
| 127 | 0.67 | XGBoost | 0.984 | 0.955 | 0.417 | 0.991 | 0.357 | 0.993 | 0.646641 |

**Supplemental Table 1. Performance of Multiple MASLD AI Models Trained on KNN-Imputed Missing Data.** H_mean_, harmonic mean.


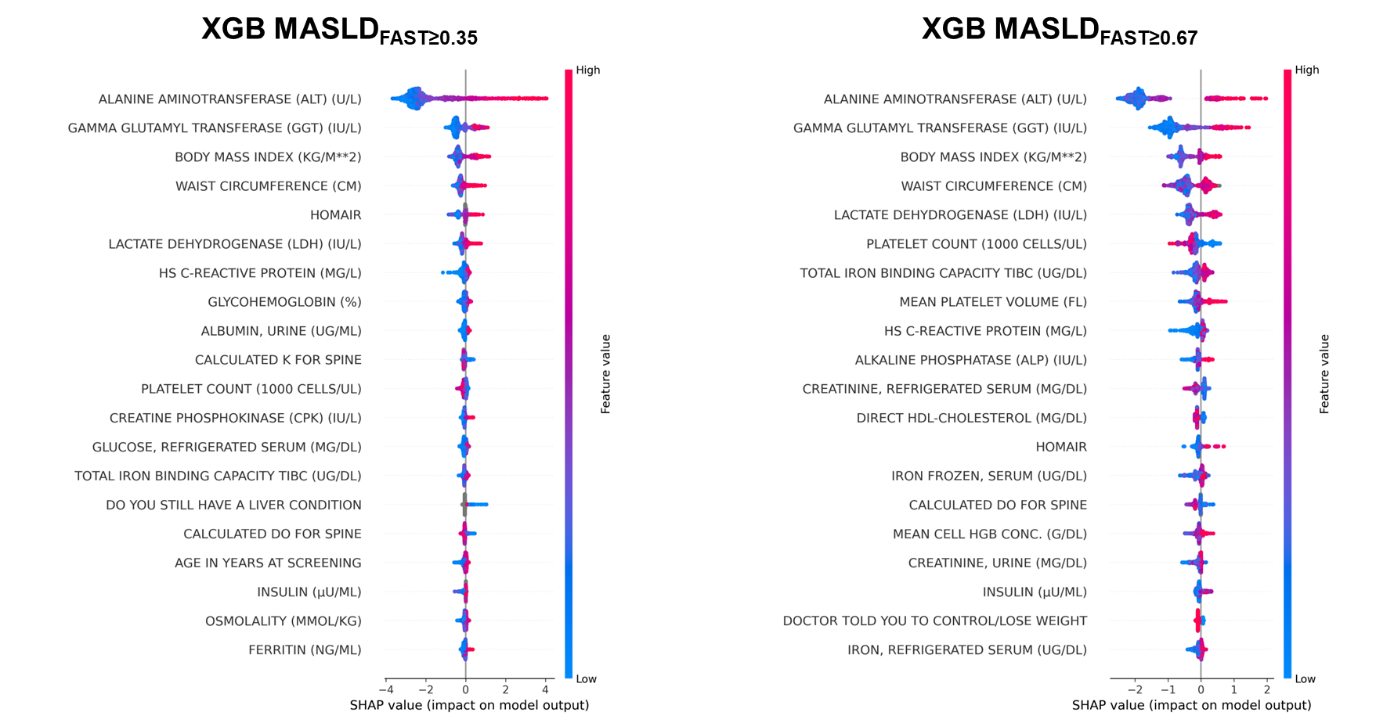


**Supplemental Figure 5. Cohort-Level Contributions from Each Predictor in XGB MASLD Model Predictions.** Beeswarm plots of distribution of SHAP values for all 127 predictors, for XGB MASLD_FAST≥0.35_ (left) and XGB MASLD_FAST≥0.67_ (right). SHAP = 0 (solid line) correspond to binary prediction label (high-risk MASLD likely if SHAP > 0 and vice versa), respectively.


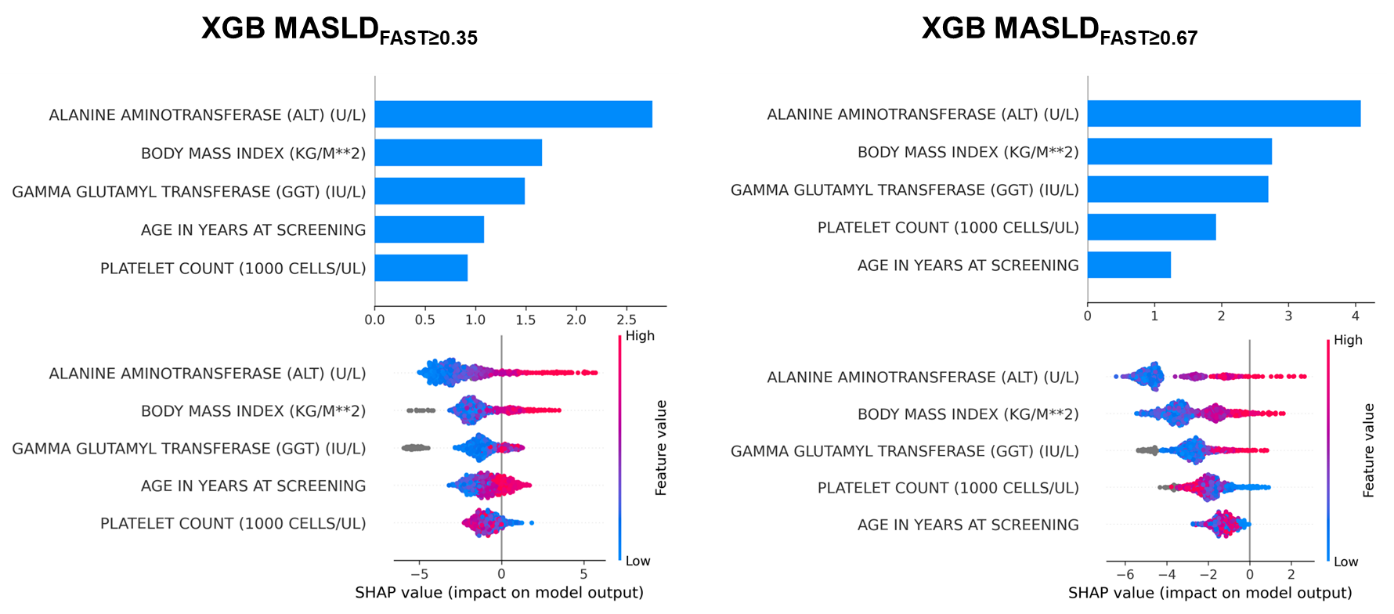


**Supplemental Figure 6. Cohort-Level Contribution from Each Predictor in XGB MASLD Model Predictions.** Bar plots (top) and beeswarm plots (bottom) of the mean absolute value of each predictor’s SHAP and distribution of SHAP values, respectively, for XGB MASLD_FAST≥0.35_ (left) and XGB MASLD_FAST≥0.67_ (right). SHAP = 0 (solid line) correspond to binary prediction label (high-risk MASLD likely if SHAP > 0 and vice versa), respectively.


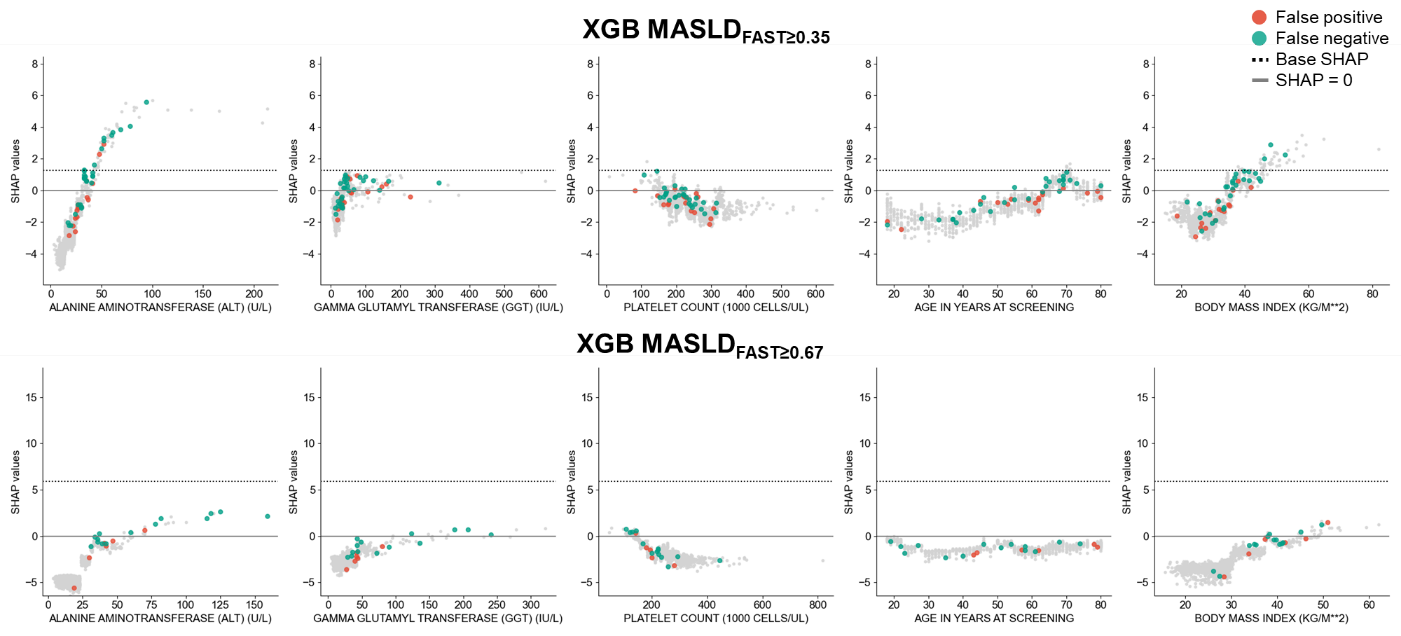


**Supplemental Figure 7. Contribution from Each Predictor in XGB MASLD Model Predictions.** Scatterplots of SHAP contribution and values for the five clinical predictors used to train XGB MASLD_FAST≥0.35_ (top) and XGB MASLD_FAST≥0.67_ (bottom). False positive (orange), false negative (green), and correct predictions (gray) are labeled. The model’s base SHAP (dashed line), and SHAP = 0 (solid line) correspond to the model’s baseline bias and binary prediction label (high-risk MASLD likely if SHAP > 0 and vice versa), respectively.
