# Supplementary material for: An explainable machine learning model for prediction of high-risk nonalcoholic steatohepatitis
Source: Sci Rep. 2024 Apr 13;14:8589. doi: 10.1038/s41598-024-59183-4 (PMC11016071; doi:10.1038/s41598-024-59183-4)
Supplement: Supplementary file 4 — Supplementary Information 4. [file 41598_2024_59183_MOESM4_ESM.docx]

**Supplemental Methods**

**1. NHANES Data Processing**

The data processing for this study involved multiple steps to ensure the quality and clinical relevance of the data.

**1.1. Vibration-Controlled Transient Elastography (VCTE)**

We excluded subjects with missing values for median stiffness (E), kilopascals (kPa) and median controlled attenuation parameter (CAP), in decibels per meter (dB/m). This step was important to calculate the FibroScan®-AST (FAST) score. Furthermore, we applied a condition to include only subjects with a completed elastography exam, at least 10 measures attempted with the final wand, and a stiffness interquartile range less than 30.

**1.2. FibroScan®-AST (FAST) score**

The FAST score was calculated using the equation by Newsome et al^1^. We used the 0.35 and 0.67 cutoff values to train eXtreme Gradient Boosting (XGBoost) models to predict high-risk MASLD at different FAST cutoff points.

**1.3. Demographics**

Individuals below 18 years of age, individuals with a positive hepatitis B surface antibody, hepatitis C antibodies, or more than 1 or 2 daily average alcoholic beverages in the past 12 months for women and men, respectively, were excluded.

**1.4. Serologic Biomarkers**

We calculated serologic biomarkers and scores including Homeostatic Model Assessment for Insulin Resistance (HOMA-IR), Fibrosis-4 Index (FIB4), NAFLD Fibrosis Score (NFS), BMI-AAR-T2DM (BARD), Aspartate Aminotransferase to Platelet Ratio Index (APRI) with the formulas in **Supplemental Methods Table 1**. To calculate specific serologic biomarkers for all subjects, we used K-nearest neighbor imputation (k=5) to impute missing values for age, gender, HbA1c, history of diabetes mellitus, BMI, waist circumference, AST, ALT, GGT, insulin, platelet count.

| **Biomarker** | **Formula** |
| --- | --- |
| HOMA-IR | $Insulin*Fasting Glucose/405$ |
| FIB4 | $(Age*AST)/(Plt*\sqrt{ALT})$ |
| NFS | $Age*0.037+BMI*0.094+DM*1.13+AAR*0.99-Plt*0.013-Albumin*0.66$ |
| BARD | $1*\left( BMI\geq28 \right)+2*\left( AAR\geq0.8 \right)+1*DM$ |
| APRI | $(AST*40)/Plt$ |

Table 1: Serologic Biomarkers and Formulas.

**2. XGBoost Model Development**

**2.1. Hyperparameter Optimization**

In this work, we developed a model using the XGBoost algorithm^2^, optimized through hyperparameter tuning with Hyperopt^3^. The hyperparameter search space **(Supplemental Methods Table 2)** included the maximum depth of the trees, ranging from 3 to 20, and the minimum child weight, with a uniform quantized distribution between 0 and 10. The subsample ratio and the column sample by tree ratio were set with uniform distributions between 0.4 to 1 and 0.3 to 1, respectively. The learning rate followed a log-uniform distribution log_10_(0.005) to log_10_(0.3), while the number of estimators was selected from a range of 50 to 1500, increasing in increments of 50. The gamma value, with a range of 0 to 10 in increments of 0.5, along with regularization lambda and alpha parameters, both ranging uniformly from 0 to 5, were also tuned. Additionally, the tree method was fixed to 'hist', and the device was set to utilize CUDA. Early stopping rounds were set to 5.

The optimization process involved adjusting the scale positive weight based on the training data and employing a specific objective function. This function accounted for metrics such as sensitivity, specificity, positive predictive value (PPV), and negative predictive value (NPV), aiming to optimize the model based on the harmonic mean of these metrics. The best parameters found from this optimization were then used to train the model. The trained model was subsequently evaluated on a test dataset, with metrics including the Area Under the Receiver Operating Characteristic Curve (AUROC), accuracy, sensitivity, specificity, PPV, and NPV being calculated. We generated a table with the actual labels, predicted labels, and predicted probabilities from the test set evaluation.

| **Parameter** | **Range/value** | **Distribution** | **Description** |
| --- | --- | --- | --- |
| Maximum depth | 3 to 20 | Uniform | The maximum depth of a tree. Deeper trees can model more complex patterns but might lead to overfitting. |
| Minimum child weight | 0 to 10 | Uniform quantized | The minimum sum of instance weight (hessian) needed in a child. Used to control overfitting. |
| Subsample ratio | 0.4 to 1 | Uniform | The fraction of samples to be used for fitting each tree. A smaller value prevents overfitting but can underfit. |
| Column sample by tree | 0.3 to 1 | Uniform | The fraction of features to be used for each tree. A technique to prevent overfitting. |
| Learning rate | log_10_(0.005) to log_10_(0.3) | Log-uniform | Determines the step size at each iteration while moving toward a minimum of a loss function. A smaller learning rate requires more boosting rounds. |
| Number of estimators | 50 to 1500 (increments of 50) | Incremental | The number of boosting rounds or trees to build. More trees can lead to more accurate models but may lead to overfitting. |
| Gamma | 0 to 10 (increments of 0.5) | Uniform quantized | A node is split only if the split gives a positive reduction in the loss function. Higher values lead to fewer splits. |
| Regularization lambda | 0 to 5 | Uniform | L2 regularization term on weights. It encourages smaller weights, thus simpler models, and less overfitting. |
| Regularization alpha | 0 to 5 | Uniform | L1 regularization term on weights. It can lead to sparse models (with feature selection). |
| Tree method | hist | (fixed value) | The algorithm used for constructing the trees. 'Hist' is efficient for large datasets. |
| Device | cuda | (fixed value) | The type of hardware to run the model. 'cuda' indicates GPU acceleration. |
| Early stopping rounds | 5 | (fixed value) | The training stops if the validation metric does not improve for a given number of boosting rounds. Helps in preventing overfitting. |

Table 2. Hyperparameter search space.

**References**

1. Newsome, P. N. *et al.* FibroScan-AST (FAST) score for the non-invasive identification of patients with non-alcoholic steatohepatitis with significant activity and fibrosis: a prospective derivation and global validation study. *Lancet Gastroenterol. Hepatol.* **5**, 362–373 (2020).

2. Chen, T. & Guestrin, C. XGBoost: A Scalable Tree Boosting System. in *Proceedings of the 22nd ACM SIGKDD International Conference on Knowledge Discovery and Data Mining* 785–794 (ACM, New York, NY, USA, 2016). doi:10.1145/2939672.2939785.

3. Bergstra, J., Yamins, D. & Cox, D. Making a Science of Model Search: Hyperparameter Optimization in Hundreds of Dimensions for Vision Architectures. in *Proceedings of the 30th International Conference on Machine Learning* (eds. Dasgupta, S. & McAllester, D.) vol. 28 115–123 (PMLR, Atlanta, Georgia, USA, 2013).
